# *Batrachochytrium salamandrivorans* is the predominant chytrid fungus in Vietnamese salamanders

**DOI:** 10.1038/srep44443

**Published:** 2017-03-13

**Authors:** Alexandra E. Laking, Hai Ngoc Ngo, Frank Pasmans, An Martel, Tao Thien Nguyen

**Affiliations:** 1 Department of Pathology, Bacteriology and Avian Diseases, Faculty of Veterinary Medicine, Ghent University, Salisburylaan 133, 9820 Merelbeke, Belgium; 2 Vietnam National Museum of Nature, Vietnam Academy of Science and Technology, 18 Hoang Quoc Viet, Cau Giay, Hanoi, Vietnam

## Abstract

The amphibian chytrid fungi, *Batrachochytrium dendrobatidis (Bd*) and *B. salamandrivorans (Bsal*), pose a major threat to amphibian biodiversity. Recent evidence suggests Southeast Asia as a potential cradle for both fungi, which likely resulted in widespread host-pathogen co-existence. We sampled 583 salamanders from 8 species across Vietnam in 55 locations for *Bsal* and *Bd*, determined scaled mass index as a proxy for fitness and collected environmental data. *Bsal* was found within 14 of the 55 habitats (2 of which it was detected in 2013), in 5 salamandrid species, with a prevalence of 2.92%. The globalized pandemic lineage of *Bd* was found within one pond on one species with a prevalence of 0.69%. Combined with a complete lack of correlation between infection and individual body condition and absence of indication of associated disease, this suggests low level pathogen endemism and *Bsal* and *Bd* co-existence with Vietnamese salamandrid populations. *Bsal* was more widespread than *Bd*, and occurs at temperatures higher than tolerated by the type strain, suggesting a wider thermal niche than currently known. Therefore, this study provides support for the hypothesis that these chytrid fungi may be endemic to Asia and that species within this region may act as a disease reservoir.

Globalization has led to the emergence of infectious diseases that threaten biodiversity and contribute to the current 6^th^ mass extinction, a recent example being amphibian chytrid fungi: *Batrachochytrium dendrobatidis (Bd*), which has caused declines and extinctions within Anura, Urodela and Gymnophiona throughout many parts of the world^1^,^2^,^3^,^4^,^5^,^6^,^7^ and *B. salamandrivorans (Bsal*), which is causing massive declines in salamander populations in Europe^8^,^9^. *Bsal* appears to be restricted to urodelan hosts, causing erosive skin lesions with subsequent death in diseased individuals, and the type strain has lower thermal growth tolerance limits than its sister species *Bd*^8^. A recent screening for *Bsal* revealed the presence of the fungus during disease outbreaks in wild salamander populations within the Netherlands, Germany and Belgium^9^, and in captive animals from the UK and Germany^10^,^11^. The presence of *Bsal* in several urodelan species in Thailand, Vietnam and Japan, coupled with clinical infection trials indicating three Asian salamander species were capable of persisting with the infection or clearing the infection completely, gave rise to the hypothesis that *Bsal* is endemic to eastern Asia and the salamander species inhabiting this area may act as a reservoir for the fungus^12^. This hypothesis has been proposed for *Bd* within Asia as well, due to the fact that a high diversity of lineages is found in eastern Asia^13^,^14^ and, throughout this region *Bd* has a low prevalence with no signs of disease outbreak in many amphibian species^15^,^16^,^17^,^18^,^19^,^20^. A reservoir, in relation to disease, has been defined as “a passive host or carrier that harbors pathogenic organisms without injury to itself and serves as a source from which other individuals can be infected”^21^. In regards to *Bsal* and *Bd*, we thus predict suitable reservoirs to sustain the fungus in the absence of mortality and disease events that negatively affect the host populations. Here we conducted the first large scale screening for *Bsal*, along with *Bd*, in Southeast Asia to test this hypothesis. In order to do this, we sampled 583 salamanders belonging to 8 urodelan species of the salamandrid genera *Paramesotriton* and *Tylototriton* across 55 localities (ponds or streams) in Vietnam to test for the presence of *Bsal* and *Bd.* We then looked into associations of pathogen prevalence and load with potentially influential environmental factors and the presence of associated disease.

## Results

Across 11 provinces located in northern Vietnam, 44 ponds and 11 streams were sampled and 583 individuals from 8 species, belonging to the genera *Paramesotriton* and *Tylototriton*, were sampled for the presence of *Bsal* and *Bd* and their body condition determined (Table 1). A total of 17 individuals belonging to 5 species (*T. vietnamensis, T. ziegleri, T. asperrimus, P. deloustali* and *P.* sp.) tested positive for *B. salamandrivorans* resulting in an overall prevalence of 2.92% (95% CI: 1.80%, 4.65%). Two of the three species which did not contain positive individuals had small sample sizes (*T. anguliceps* (8), *T.* sp. (17) and *P. guanxiensis* (54)) and as the prevalence is low within this area more samples need to be gathered to definitively determine if these populations contain infected individuals. The average prevalence of the 14 positive sites was 17.56% (SD ± 25.27, 95% CI: 3.06%, 32.24%). Four individuals of *T. ziegleri*, in one pond, tested positive for *Bd* resulting in an overall prevalence of 0.69% (95% CI: 0.20%, 1.83%) and a prevalence of 30.77% (95% CI: 12.35%, 57.96%) within the positive pond. The *Bd* samples amplified with the global pandemic lineage (*Bd*GPL) probes indicating a *Bd*GPL-like lineage.

*Bsal* infection prevalence did not differ between provinces or species (Fisher’s exact tests, P = 0.34 and P = 0.25, respectively). There were no correlations between infection intensity of *Bsal* or *Bd* and scaled mass index (SMI) for any of the eight species (Spearman’s correlations < ±0.18). Elevation, province, species, scaled mass index, water temperature and ambient temperature were not significant predictors of infection intensity for *Bsal* (Generalized linear model, P > 0.05).

## Discussion

**Figure 1:**
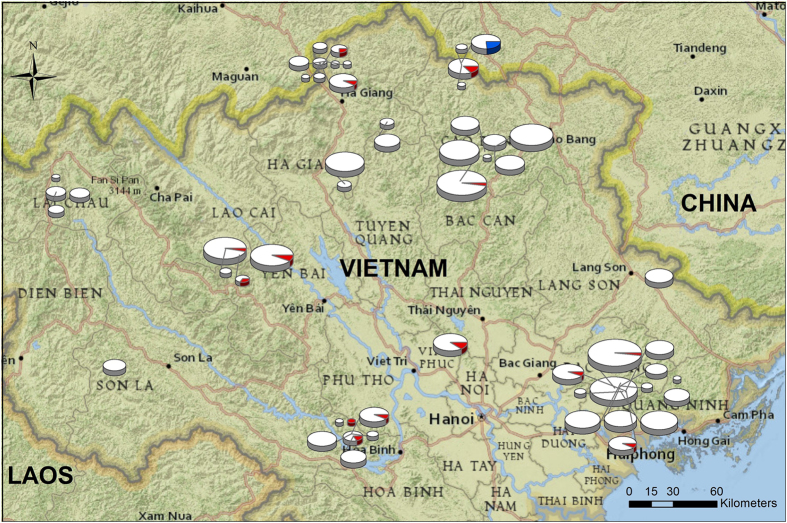
Map of sampling locations within Northern Vietnam. The size of the pie charts corresponds to the sample size ranging from 1 to 44 individuals. The pie chart indicates the prevalence of *Bsal* (Red) or *Bd* (Blue) within that population. This map was generated in ArcMap 10.1 (http://www.esri.com/software/arcgis/arcgis-for-desktop).

Our study provides the first large scale screening for *Batrachochytrium salamandrivorans (Bsal*) within Southeast Asia, specifically Vietnam. We found that *Bsal* was much more widespread throughout salamander populations in Vietnam than its sister species *B. dendrobatidis (Bd*), occurring in 14 of the 55 locations compared to the one pond in which *Bd* was detected (Fig. 1), corroborating the predilection of *Bsal* for urodelan hosts^12^ and providing further evidence for a *Bd* prevalence that is low throughout Asia^15^,^16^,^17^,^18^,^19^,^22^. We did not find any signs of *Bsal* associated disease such as decreased body condition, lesions, or death in the infected populations. The overall low prevalence for both *Bsal* (2.92%) and *Bd* (0.69%), despite presence of *Bsal* in Vietnamese salamander populations at least since 2013^12^, combined with the absence of disease signs indicates endemism of both species of chytrid fungi within Vietnamese salamander populations, adds support to the hypothesis that South East Asian salamanders constitute a *Bsal* reservoir. Similarly low prevalence but widespread occurrence has been demonstrated for *Bd* in regions where *Bd* is considered endemic in the absence of epidemic disease^13^,^23^,^24^. If compensated, co-existence does not exclude an actual cost of *Bsal* on the Vietnamese salamander populations, as has been demonstrated for *Bd*^23^,^25^, especially given the potential of both pathogens to cause juvenile mortality under experimental conditions^12^,^22^.

Through the use of lineage specific qPCR, we found that the *Bd* we detected amplified as global pandemic lineage (*Bd*GPL) indicating it could be the true *Bd*GPL, endemic or non-endemic *Bd*GPL-like, or a hybrid of an endemic and *Bd*GPL cross^26^. *Bd*GPL is associated with all of the known epizootic events and has experimentally been shown to be more virulent than other lineages^27^. As *Bd* seems to occur in an endemic state within Asia and our study site of Vietnam, it is likely an endemic *Bd*GPL-like lineage, further testing should be done to determine the strain as well as the virulence of the *Bd* within Vietnam.

**Figure 2:**
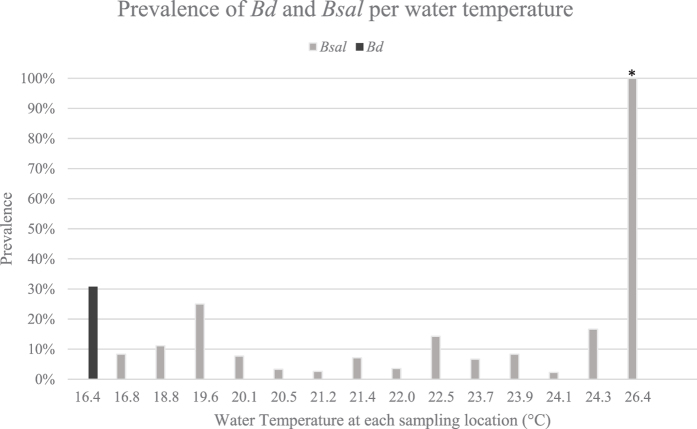
Prevalence of *Bd* and *Bsal* positive salamanders at each positive location (pond or stream), and its corresponding water temperature. *Only one individual was sampled at this location.

The thermal optimum for the type strain of *Bsal* is 15–20 °C^28^, whereas the majority of infected individuals we found in Vietnam were in ponds or streams with water temperatures between 20–25 °C, even reaching 26.43 °C in one positive location (Fig. 2). This might have serious implications as differential thermal preferences of strains could significantly expand the invasive niche if the fungus is vectored into a naive region (Yap *et al*.^29^).

In conclusion, due to the low prevalence of *Bsal* and *Bd*GPL and lack of correlation between any of the environmental or physical parameters, we provide evidence supporting the hypothesis that these chytrid fungi may be endemic to Asia and that species within this region may act as a disease reservoir for naive regions. We found that *Bsal* is much more widespread within northern Vietnam, occurring at temperatures higher than those tolerated by the fungus’ type strain, which suggests a wider thermal niche than currently known. Further screening should be conducted to determine the range of *Bsal* and which species carry the fungus. These results reinforce the notion that there should be strict biosecurity measures in place, in order to prevent the further spread of *Bsal* and avoid a global impact similar to that of *Bd.*

## Methods

### Study Species

Eight salamander species were sampled for the presence of *B. salamandrivorans: Tylototriton vietnamensis* (207 individuals), *T. ziegleri* (110 indv.), *T.* sp. (17 indv.), *T. anguliceps* (8 indv.), *T. asperrimus* (46 indv.), *Paramesotriton deloustali* (80 indv.), *P. guangxiensis* (54 indv.) and *P.* sp. (61 indv.). Nets were utilized to capture salamanders within the stream or pond. Each salamander was weighed, using a digital scale (MS-Series,G&G GmbH), and measured for snout to vent and tail length, using a 0–150 mm digital caliper (Fixpoint). A scaled mass index was calculated following the method developed by Peig and Green^30^.

### Data collection

Since the *Bsal* type strain has a low tolerance for high environmental temperatures, these were predicted to drive *Bsal* infection. Therefore, at each pond or stream water temperature and ambient temperature were measured using an HHC201 thermocouple thermometer, with a hermetically sealed thermocouple (OMEGA^®^). Prevalence and infection loads of *Bsal* and *Bd* in the captured amphibians was determined by collecting non-invasive skin swabs^29^. All methods were carried out in accordance with Vietnamese guidelines and regulations. Experimental procedures were approved and research was carried out under permits obtained for each province of Vietnam through the corresponding provincial government and the Ministry of Agriculture and Rural Development (MARD) (Lang son: 224/SNgV-LS, Bac Giang: 866/UBND-XD, Quang Ninh: 1781/UBND-MT, Cao Bang: 998/UBND-NC, Lai Chau: 663/UBND-NC, Hoa Binh: 1444/VPUBND-TH, Ha Giang: 107/SNgV-NVLS, Bac Kan: 1293/UBND-NV, Vinh Phuc: 104/BTTNVN, Lao Cai: 1887/UBND-NC and Son La: 304/BTTNVN).

### Sample processing

DNA was extracted from the swabs using 100 μl of Prepman Ultra DNA extraction buffer. Extracted DNA samples were diluted 1/10 with HPLC water to reduce PCR inhibition^31^ and then stored at −20 °C until processing. Samples were processed using the *B. salamandrivorans* and *B. dendrobatidis* specific duplex real-time PCR procedures described by Blooi *et al*.^32^ on CFX96 real-time system (Bio-Rad Laboratories, Hercules, CA). Real-time PCR results (genomic equivalents (GE) of *B. salamandrivorans* and *B. dendrobatidis* zoospores) were corrected for the applied dilution factor. Lineage specific qPCR was conducted utilizing Taqman MGB probes that are able to discriminate single nucleotide polymorphisms in the *Bd* mitochondrial genome which are diagnostic for the globalized pandemic lineage (*Bd*GPL), following the protocol of Bletz *et al*.^26^.

### Data analysis

The modified Wald method used to calculate the 95% CI for proportion of infected individuals. Fisher exact tests were used to compare prevalence between species and provinces. We examined how GE load was affected by species, SMI, SVL, water temperature and elevation using generalized linear models (Gaussian functions) and spearman correlations in R (version 3.2.3), due to the non-normal distribution of the data.

## Additional Information

**How to cite this article**: Laking, A. *et al. Batrachochytrium salamandrivorans* is the predominant chytrid fungus in Vietnamese salamanders. *Sci. Rep.* **7**, 44443; doi: 10.1038/srep44443 (2017).

**Publisher's note:** Springer Nature remains neutral with regard to jurisdictional claims in published maps and institutional affiliations.

## Figures and Tables

**Table 1:** *Bd* and *Bsal* prevalence per province/district, indicating the number of ponds or streams sampled, the species found, the total number of animals sampled, the number of *Bd* and *Bsal* positive individuals and the corresponding prevalences.

Province, District	Sampling locations	Species	Water temperature range (°C)	Animals swabbed	Bsal positive	Bd positive	Bsal prevalence	Bd prevalence
**Bac Giang**	11 Ponds	*T. vietnamensis*	22.7–24.8	184	3	0	1.6%	0.0%
**Qiang Ninh**	2 Ponds	*T. vietnamensis*	23.8–24.9	11	0	0	0.0%	0.0%
**Lang Son**	1 Pond	*T. vietnamensis*	31.9	12	0	0	0.0%	0.0%
**Cao Bang**, **Bao Lac**	4 Ponds	*T. ziegleri*	16.07–22.53	30	2	4	6.7%	13.3%
**Cao Bang**,	4 Streams	*P. guanxiensis*	20.6–22.97	54	0	0	0.0%	0.0%
**Nguyen Binh**	1 Pond	*T. ziegleri*	19.57	8	0	0	0.0%	0.0%
**Ha Giang**, **Bac Me**	2 Ponds	*T. ziegleri*	18.7–19.7	13	0	0	0.0%	0.0%
**Ha Giang**, **Quan Ba**	9 Ponds	*T. ziegleri*	16.37–20.73	33	2	0	6.1%	0.0%
**Ha Giang**, **Bac Quang**	2 Ponds	*T. ziegleri*	21.6–23.37	26	0	0	0.0%	0.0%
**Lai Chau**	4 Ponds	*T.* sp.	18.53–23.9	17	0	0	0.0%	0.0%
**Son la**	1	*T. anguliceps*	20.6	8	0	0	0.0%	0.0%
**Hoa Binh**	7 Ponds	*T. asperrimus*	20.07–26.43	46	3	0	6.5%	0.0%
**Bac Kan**	2 Streams	*P. deloustali*	21.2–22.47	62	1	0	1.6%	0.0%
**Lao Cai**	4 Streams	*P.* sp.	20.5–22.07	61	4	0	6.6%	0.0%
**Tam Dao**	1 Stream	*P. deloustali*	18.8	18	2	0	11.1%	0.0%
**Total**	**55**	**8 species**	**16.37–31.9**	**583**	**17**	**4**	**2.9%**	**0.7%**
